# The CD4/CD8 ratio is associated with T lymphocyte functions in long-term virally suppressed patients with HIV

**DOI:** 10.1186/s12879-025-10469-6

**Published:** 2025-01-17

**Authors:** Qing Xiao, Fengting Yu, Liting Yan, Xiaojie Lao, Xuelei Liang, Hongxin Zhao, Liuyue Zhai, Zailin Yang, Xiaomei Zhang, Yao Liu, Fujie Zhang

**Affiliations:** 1 Beijing Ditan Hospital, Capital Medical University, Beijing, 100015 People’s Republic of China; 2 Chongqing Key Laboratory of Translational Research for Cancer Metastasis and Individualized Treatment, Department of Hematology-Oncology, Chongqing University Cancer Hospital, Chongqing, China; 3 Infectious Disease Department, Sichuan Provincial People’s Hospital, University of Electronic Science and Technology of China, Chengdu, China

**Keywords:** Human Immunodeficiency Virus (HIV), CD4/CD8 ratio, Antiviral therapy (ART), T lymphocytes, Mitochondria

## Abstract

**Objective:**

Long-term management of people living with HIV (PLWHs) often relies on CD4^+^ T cell counts for assessing immune recovery, yet a single metric offers limited information. This study aimed to explore the association between the CD4/CD8 ratio and T lymphocyte activities in PLWHs.

**Methods:**

125 PLWHs and 31 HIV-uninfected controls (UCs) were enrolled and categorized into four groups based on their CD4/CD8 ratios: extremely low ratio (ELR) group: 0.4 < CD4/CD8; low ratio (LR) group: 0.4 ≤ CD4/CD8<0.7; medium ratio (MR) group: 0.7 ≤ CD4/CD8<1; high ratio (HR) group: CD4/CD8 ≥ 1. The activation and proliferation phenotypes, mitochondrial functions, and inflammatory indexes of CD4^+^ T cells and CD8^+^ T cells were measured, and correlations between the CD4/CD8 ratio and T cell functions were analyzed.

**Results:**

T cell activation and proliferation were significantly elevated in the ELR group compared to UCs. However, the ELR group had a larger proportion of T cells with lipid peroxidation, mitochondrial lipid reactive oxygen species (ROS), and mitochondrial membrane potential (MMP) abnormalities compared to the other groups. As the CD4/CD8 ratio increased, mitochondrial lipid peroxidation damage decreased and MMP was restored. Additionally, the ELR group had more inflammatory markers in CD4^+^ T cells. Correlation analysis revealed that the CD4/CD8 ratio was associated with multiple T cell functions, and its correlation coefficient with mitochondrial function was higher than that of CD4^+^ T cell count.

**Conclusion:**

The CD4/CD8 ratio is closely related to T lymphocyte functions and is significantly superior to the CD4^+^ T cell count in reflecting the mitochondrial lipid peroxidation level and mitochondrial functions within T lymphocytes.

## Introduction

HIV infection is characterized by immunological hyperactivation, with an increase in CD8^+^ T cells and a decrease in CD4^+^ T lymphocytes [1]. Despite effective antiretroviral therapies (ART), people living with HIV (PLWHs) have a higher incidence of acquired immune deficiency syndrome (AIDS) and non-AIDS-related illnesses compared to healthy, HIV-uninfected individuals [2]. In some regions, the life expectancy of PLWHs on ART is lower than that of HIV-negative populations [3], which may be due to factors, such as viral reservoir persistence, intestinal flora disorders, and sustained inflammation [1, 4]. The morbidity and mortality of AIDS are associated with T cell inflammation and chronic immunological activation, and biomarkers of these persist in PLWHs even with long-term, efficient ART [5].

Traditionally, the patient’s response to therapy has been evaluated by monitoring viral load and CD4^+^ T cell count during HIV infection. With effective ART, most patients maintain CD4^+^ T cell counts above 200 cells/µL, reducing monitoring frequency [6, 7]. Guidelines suggest surveillance every 6–12 months for patients with CD4^+^ T cell counts above the risk of opportunistic infections [8]. However, immune activation often persists and CD8^+^ T cell levels rarely return to normal after ART, despite effective viral suppression and CD4^+^ T cell count recovery [9]. Currently, microbial translocation and residual HIV replication are recognized as key factors in ongoing immunological activation and inflammation [10].

At present, the main indices for evaluating immune function recovery in PLWHs include the recovery of CD4^+^ T cell number and function, as well as immune activation state and lymph node structure repair, although there is no unified standard [11]. In healthy individuals, the CD4/CD8 ratio reflects immune status and is correlated with age and ethnicity [12, 13]. As HIV research has advanced, the CD4/CD8 ratio has been found to be an important predictor of immune dysfunction and disease progression in HIV patients. HIV infection often leads to a reversal of the CD4/CD8 ratio [14], and ART may restore it, but it rarely exceeds one, especially in those with delayed treatment [14, 15]. When PLWHs have a CD4^+^ T cell count above 500 cells/µL and an undetectable viral load, their reversed CD4/CD8 ratios can persist for a long time [16, 17]. A high CD8^+^ T cell count is a reason for the failure of CD4/CD8 ratio normalization [9, 18], and a low CD4/CD8 ratio is inversely correlated with morbidity and death risk [16, 17, 19].

Immunological senescence, activation, and inflammatory markers are associated with a decrease in the CD4/CD8 ratio [14, 20]. When CD4^+^ T cell and CD8^+^ T cell numbers are within the normal range, the CD4/CD8 ratio can help detect mild immunological failure. Studies suggest that the pre-ART CD4/CD8 ratio better predicts immunological recovery [21–24], and combining CD4 and CD4/CD8 ratio aids in understanding restoration [25]. Moreover, the CD4/CD8 ratio may be a more accurate indicator of immunological failure in controlled HIV infection than CD4^+^ T cell count alone.

After four years of successful ART with full virological suppression, this study aimed to precisely define and investigate the associations between T cell activation, proliferation, mitochondrial function, and inflammatory biomarkers in a cohort of PLWHs. Specifically, we sought to understand how these factors interact with each other and with the CD4/CD8 ratio, and to determine the association of the CD4/CD8 ratio with the immunological traits in PLWHs, with the expectation of identifying the key factors influencing immune reconstitution in PLWHs.

## Materials and methods

### Patient recruitment and sample collection

The Committee of Ethics at Beijing Ditan Hospital, Capital Medical University, Beijing, China, agreed to this study (approval number: 2021-022-01). Patients who visited the Infectious Diseases Clinic of Beijing Ditan Hospital between October 2021 and March 2022, and all enrolled patients were from the antiretroviral treatment cohort, recruitment was carried out according to the following criteria: The inclusion criteria were age 30–45 years old, being on antiretroviral therapy (ART) for more than four years, and having sustained virological suppression after ART with no virological failure for at least three years. The exclusion criteria included discontinuation of ART during treatment or virological failure, presence of active opportunistic infection, combined infection with hepatitis B virus (HBV), hepatitis C virus (HCV), cytomegalovirus (CMV), syphilis or other viral infections, presence of a confirmed malignancy, and severe hepatic or renal impairment. For the uninfected controls (UCs), the inclusion criteria were age 30–45 years old (matched to the age of the PLWHs cohort) and being tested negative for HIV, HBV, and HCV infections. The exclusion criteria were any history of HIV exposure or risk behavior, the presence of any chronic illness or disease that could affect the immune system, and any current or recent (within the past 6 months) infection or illness. During the data collection process, we aimed to match the UCs with the infected group as closely as possible in terms of age and gender. For age, we attempted to keep the age difference within ± 4 years. For gender, we ensured the same gender proportion. However, due to practical limitations, the final number of the UCs was 31. EDTA-containing Vacutainer tubes were used to collect fasting blood through venipuncture (Becton Dickinson, Franklin Lakes, NJ, USA). Peripheral blood mononuclear cells (PBMCs) were separated in SepMateTM Tubes (Stemcell, Vancouver, Canada) using gradient centrifugation with Lymphoprep™ (Stemcell, Vancouver, Canada). After that, plasma was stored at -80 °C for later examination.

### Plasma HIV-1 viral load and cell counting

The Abbott Real Time HIV-1 (m2000sp) viral load test (Abbott Molecular, IL, USA), which has a lower detection limit of 40 copies/mL, was used to evaluate the plasma viral load. Conventional flow cytometry was used to determine the absolute CD4 cell counts in whole blood samples on Beckman Coulter Navios equipment (Beckman, San Jose, CA, USA).

### Flow cytometry

The phenotypic analysis was carried out using flow cytometry. These monoclonal fluorescent antibodies were utilized to assess the T cell phenotype: anti-CD3-APC-H7, anti-CD4-BB515, anti-CD4-APC, anti-CD8-AF700, anti-CD38-PE, anti-HLA-DR-BV510 (BD Biosciences, San Jose, CA). Dead cells were excluded either using viability dye FVS-BV510 or 7-AAD-percp- (BD Biosciences, San Jose, CA). Following the addition of monoclonal antibodies, events were captured using a Beckman Coulter Navios instrument (Beckman, San Jose, CA, USA) for 15 min at room temperature in the dark. Following earlier instructions, cells were fixed, surface dyed, and permeabilized using the Transcription Factor Buffer Set (BD Biosciences, San Jose, CA), and then stained intracellularly with the aforementioned intracellular antibodies in order to detect the nuclear proteins, including perforin-Percp/cy5.5, granzyme B-PE-cf594, and Ki-67-PE (BD Biosciences, San Jose, CA). The PBMCs (1 × 10^6^) were cultured in RPMI-1640 supplemented with 10% fetal bovine serum (GIBCO, USA). They were then stimulated for 4 h in 5% CO_2_ using media containing ionomycin, brefeldin-A, and phorbol 12-myristate 13-acetate (Leukocyte activation cocktail with Golgiplug; BD Biosciences). Anti-CD3 APC-H7, anti-CD4 BB515, and anti-CD8 AF700 were used to stain the surface. The Fixation/Permeabilization Kit (BD Biosciences, San Jose, CA) was then used for fixation and permeabilization, and intracellular staining was then carried out with anti-TNF-α APC, anti-IL-6 PE, and anti-INF-γ BB700. Every antigen was obtained from BD Biosciences.

### Mitochondrial function test

We determined the degree of cell damage in each group by computing the ratio of monomer to polymer and measuring the change in the mitochondrial membrane potential (MMP) based on the color change using the JC-1 fluorescent probe (Thermo Fisher, MA, USA). The amount of lipid ROS produced was measured using Liperfluor (Dojindo, Kumamoto, Japan). After incubating the PBMCs at 37℃ for 30 min in the dark with 5 µM Liperfluor dye, lipid peroxidation was assessed using 5 µM C11-BODIPY 581/591 (Invitrogen, Taufkirchen, Germany) labeling. The shift in the ratio of green to red fluorescence, which happens when a part of the C11-BODIPY fluorophore is oxidized and the fluorescence emission changes from red to green, was indicative of an increase in lipid peroxidation.

### Study Design and follow-up

This study design is a cross-sectional analysis that utilizes samples collected at a single time point from an existing antiviral cohort and a control group. The participants were included in accordance with the aforementioned inclusion and exclusion criteria, and the data collected at this single time point was comprehensive, encompassing various immunological parameters as described in the study.

### Statistical analysis

The FlowJo (version 10.8.1) was utilized for the collection and analysis of the flow cytometry data. To conduct statistical analyses, GraphPad Prism 8.0 and R 4.0.5 versions were utilized. Continuous variables were summarized using means and standard deviations, or medians and interquartile ranges. The Student’s t-test or nonparametric test (Mann-Whitney test) was used to compare continuous variables between two groups, while the ANOVA test or nonparametric test, was employed to compare continuous variables across several groups. The correlations were discovered using the Spearman rank correlation test, where r denotes the Spearman correlation coefficient. By using the *p* < 0.05, statistical significance was established.

## Results

### Clinical characteristics of the patients

A total of 31 UCs were enrolled in this study, including thirty males and one female. And 125 HIV patients on ART for more than 4 years were included, among which 119 were males and 6 were females. They were divided into four groups according to their CD4/CD8 ratio and the groupings are shown in the Table 1, and all data are collected in this table. UC group: uninfected healthy control; ELR group: extremely low ratio group, 0.4 < CD4/CD8; LR group: low ratio group, 0.4 ≤ CD4/CD8<0.7; MR group: medium ratio group, 0.7 ≤ CD4/CD8<1; HR group: high ratio group, CD4/CD8 ≥ 1.

**Table 1:** General characteristics of the study subjects

Parameters	UC group (*n* = 31)	ELR group 0.4 < CD4/CD8 (*n* = 19)	LR group 0.4 ≤ CD4/CD8<0.7(*n* = 38)	MR group 0.7 ≤ CD4/CD8<1(*n* = 37)	HR group CD4/CD8 ≥ 1(*n* = 31)
**Age (years)**					
Median	35	39	35	32	38
IQR	33–39	33–46	31–41	28–36	32–43
**Gender**					
Male	30	19	34	36	30
Female	1	0	4	1	1

### The correlation between CD4/CD8 ratio and T cell proliferation and activation

**Fig. 1:**
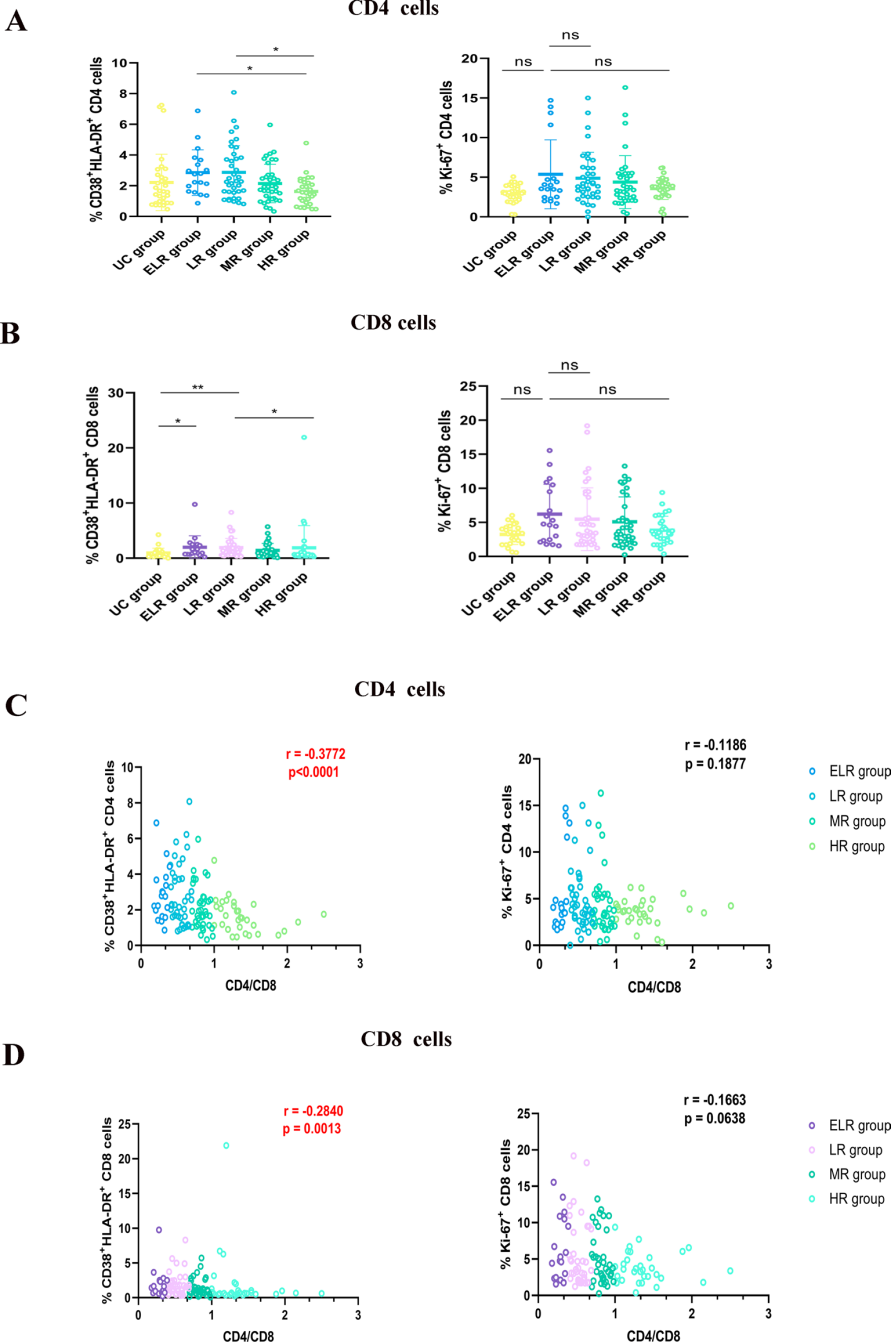
Correlation between T-lymphocyte activation and proliferation and the CD4/CD8 ratio. (**A**) Beeswarm boxplots for CD38^+^HLA-DR^+^ and Ki-67^+^ in CD4^+^ T cells, (**B**) and CD8^+^ T cells. (**C**) Analysis of the correlation between the expression of CD38^+^HLA-DR^+^ and Ki-67^+^ in CD4^+^ T cells and CD8^+^ T cells and the CD4/CD8 ratio

First, we analyzed the markers of CD4^+^ and CD8^+^T cell proliferation (Ki-67^+^) and activation (CD38^+^HLA-DR^+^) in UCs and PLWHs. As shown in Fig. 1, the CD4^+^ T cell activation and proliferation phenotypes were most pronounced in the ELR group, whereas the degree of CD4^+^ T cell activation and proliferation in PLWHs receiving ART gradually decreased with the CD4/CD8 ratio increasing. However, activated and proliferating CD8^+^ T cells differed less among groups. The degree of activation of CD4^+^ T cells and CD8^+^ T cells was found to be positively correlated with the CD4/CD8 ratio, according to correlation analysis (*r*=-0.3778, *r*=-0.2840).

### The association between CD4/CD8 ratio and T cell killing functions

**Fig. 2:**
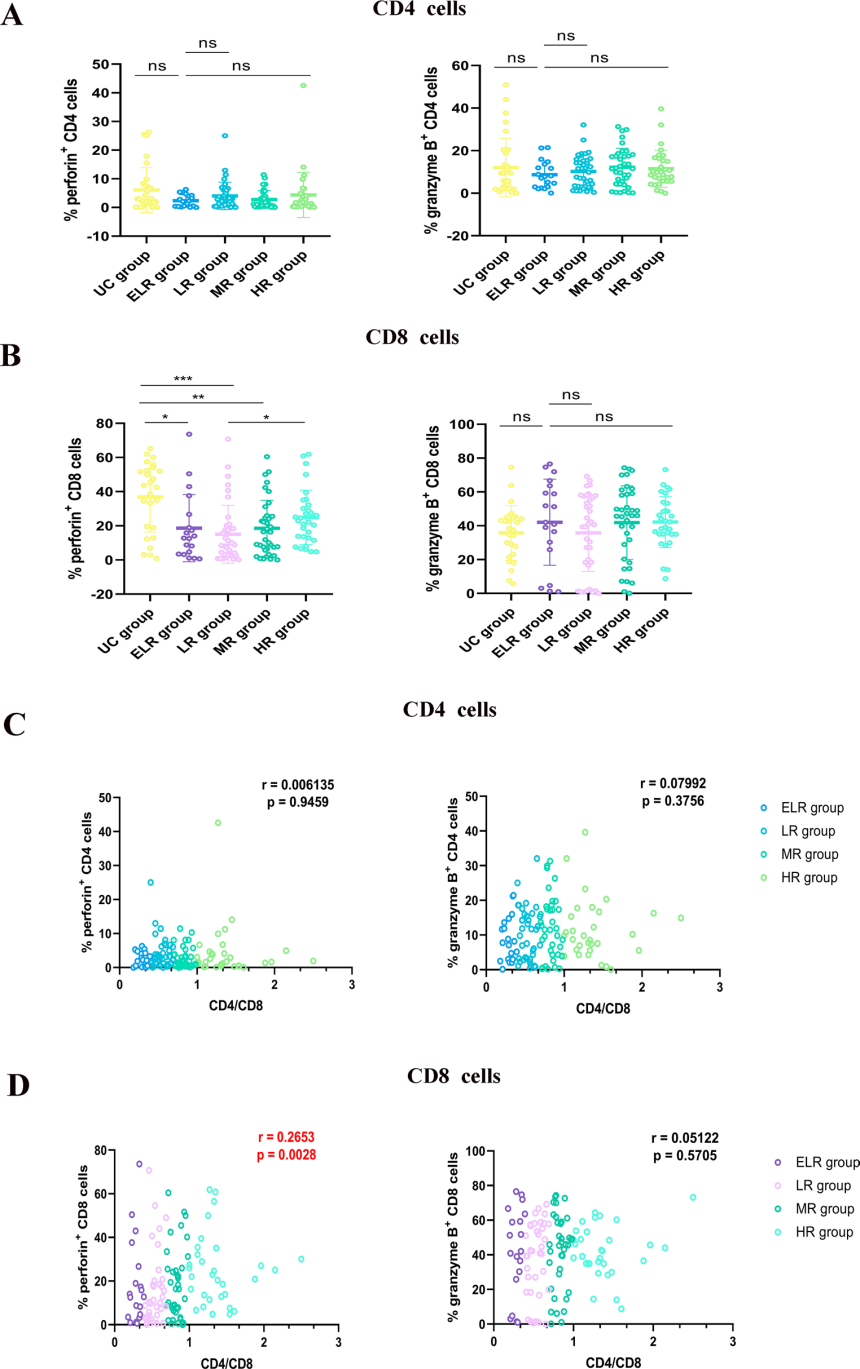
Relationship between CD4/CD8 ratio and T-lymphocyte killing functions. (**A**) Release of perforin and granzyme B secreted by CD4^+^ T cells, (**B**) and CD8^+^ T cells. (**C**) Correlation analysis of the CD4/CD8 ratio with the amount of perforin and granzyme B secreted by CD4^+^ T cells, (**D**) and CD8^+^ T cells

When we evaluated the killing capabilities of T cells, we discovered that while the synthesis of perforin in CD8^+^ T cells varied significantly among groups, the secretion of granzyme B and perforin by CD4^+^ T cells was not significantly different. Among the PLWHs groups, CD8^+^ T cells in the ELR group and the LR group produced less granzyme B. Meanwhile, with the increase of CD4/CD8 ratio, the killing function of CD8^+^ T cells in the MR group and the HR group was gradually restored, and the secretion of perforin gradually increased. Correlation analysis showed that the CD4/CD8 ratio was positively linked to the perforin secreted by CD8^+^ T cells (*r* = 0.2653) (Fig. 2).

### The relationship between CD4/CD8 ratio and T cell mitochondrial functions

**Fig. 3:**
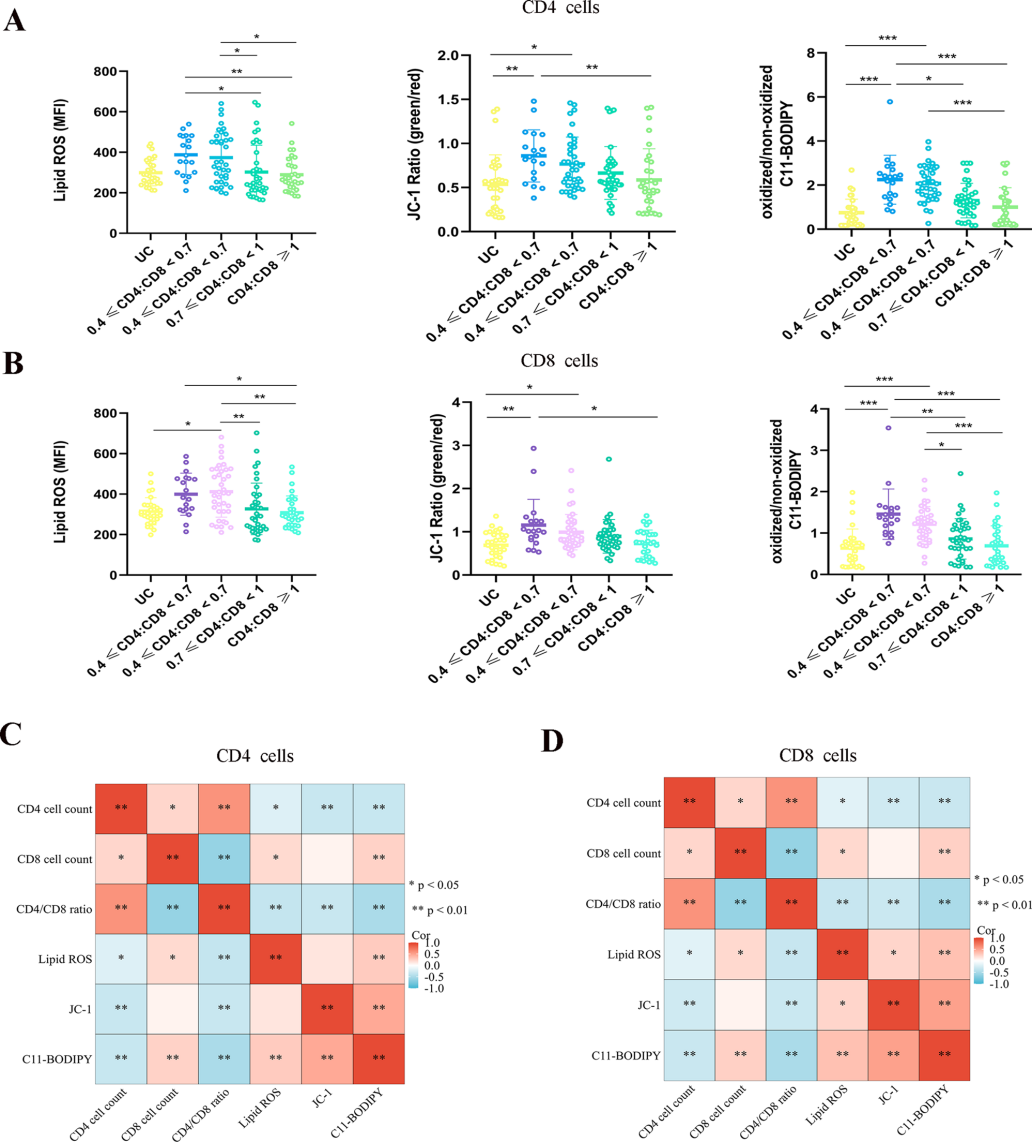
Relationship between CD4/CD8 ratio and mitochondrial functions in T-lymphocyte. (**A**) CD4^+^ T cell mitochondrial functions. (**B**) CD8^+^ T cell mitochondrial functions. (**C**) Correlation analysis of the CD4/CD8 ratio with mitochondrial functions in CD4^+^ T cells, (**D**) and CD8^+^ T cells

Since normal mitochondrial function is essential for the health of eukaryotic cells [26], whereas oxidative stress processes may cause mitochondrial structural and functional dysfunction, leading to overproduction of ROS and ultimately cell death [27]. Therefore, we employed some mitochondrial dyes to detect the degree of lipid ROS, mitochondrial membrane potential (MMP), and mitochondrial lipid peroxidation. The findings demonstrated a strong correlation between the CD4/CD8 ratio and CD4^+^ and CD8^+^ T cells’ mitochondrial functions. As the ratio of CD4/CD8 rises, the mitochondria of these PLWHs gradually produced less ROS, the damaged MMP was improved, and the degree of lipid peroxidation was reduced. Meanwhile, the results of the correlation analysis suggested that the CD4/CD8 ratio was closely related to the mitochondrial lipid ROS content, MMP, and lipid peroxide level of CD4^+^ T cells (*r*=-0.35; *r*=-0.33; *r*=-0.5), as well as to the mitochondrial function of CD8^+^ T cells (*r*=-0.34; *r*=-0.32; *r*=-0.5) (Fig. 3).

### The correlation between CD4/CD8 ratio and T cell inflammation

**Fig. 4:**
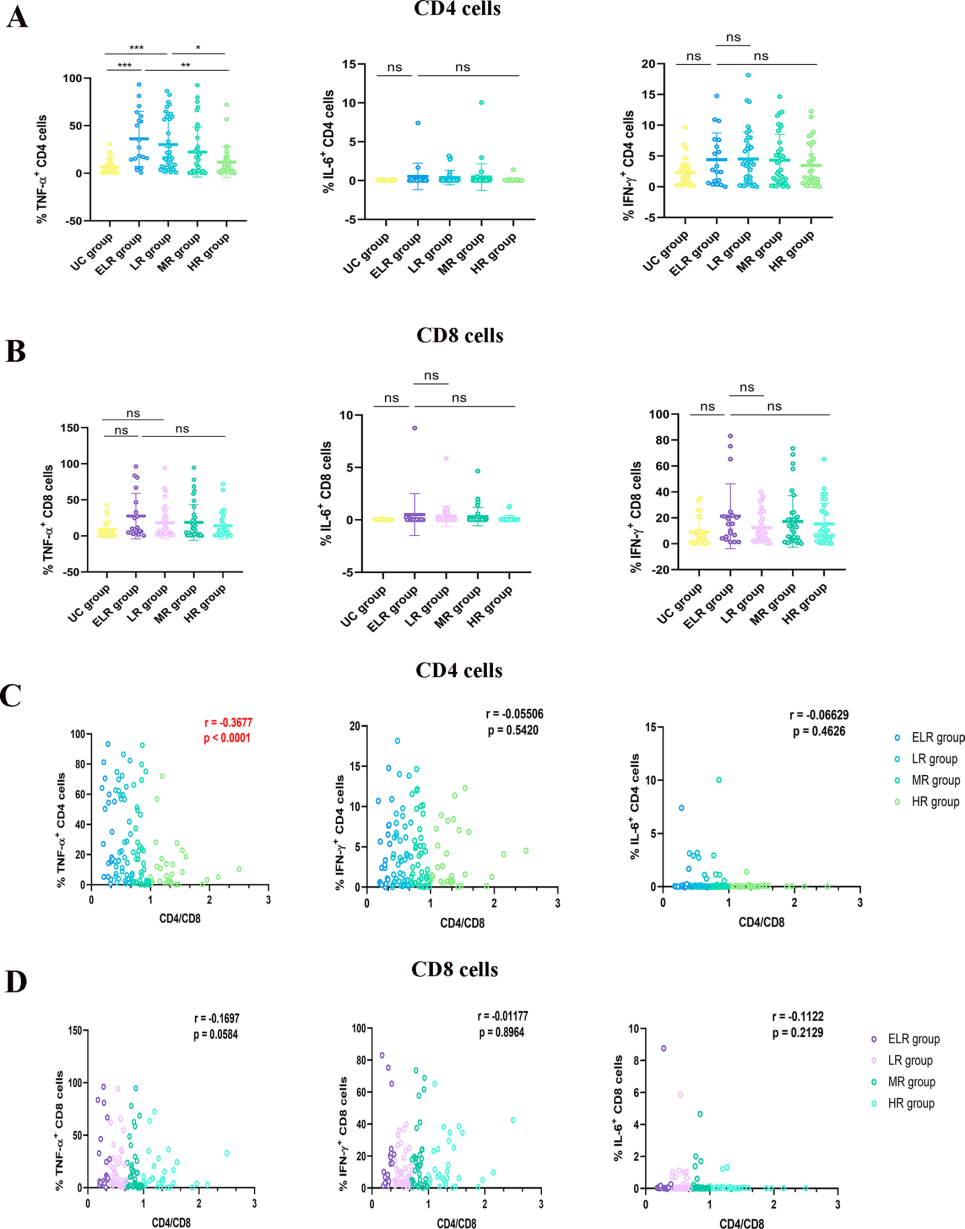
Relationship between CD4/CD8 ratio and inflammation in T-lymphocytes. (**A**) Levels of inflammation in CD4^+^ T cell, (**B**) and CD8^+^ T cell; (**C**) Correlation analysis of CD4/CD8 ratio with CD4^+^ T cell inflammation, (**D**) and CD8^+^ T cell inflammation

Inflammatory injury may be caused by dysfunctional mitochondria. We next examined the production of intracellular inflammatory markers and discovered that the ELR group had the highest levels of TNF-α released by CD4^+^ T cells. The rest of the inflammatory factors differed less and were not statistically significant among groups. With the increase of the CD4/CD8 ratio, the level of intracellular inflammation in CD4^+^ T cells gradually decreased. But the differences of cytokines in CD8^+^ T cells were not significant between each group. Next, we analysed the association between the CD4/CD8 ratio and each inflammatory index, and found a negative correlation between the CD4/CD8 ratio and the TNF-α release in CD4^+^ T cells in all patients who achieved virological suppression (*r* = -0.3677) (Fig. 4).

### The relationship of CD4 cell count, CD8 cell count, and CD4/CD8 ratio with T lymphocyte functions

**Fig. 5:**
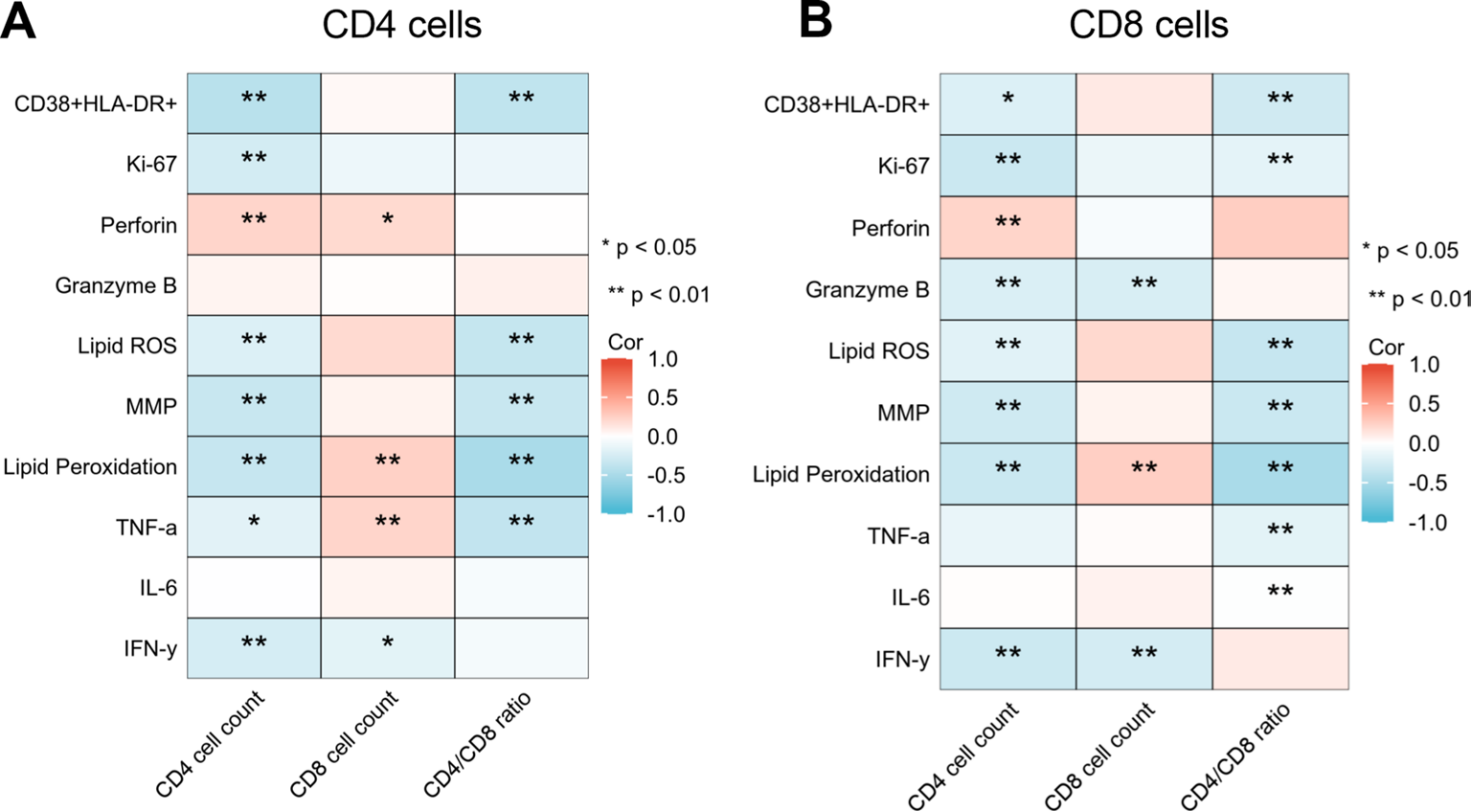
Correlation analysis of CD4^+^ T cell count, CD8^+^ T cell count, CD4/CD8 ratio with T-lymphocyte functions. (**A**) Correlation analysis of indicators in CD4^+^ T cells, (**B**) and CD8^+^ T cells

Finally, we compared the relationship between CD4^+^ T cell count, CD8^+^ T cell count, and CD4/CD8 ratio with various functional indicators of CD4^+^ T cells and CD8^+^ T cells. The results showed that both CD4^+^ T cell counts and CD4/CD8 ratios were correlated with multiple T cell functions. Among them, CD4^+^ T cell count was negatively correlated with the activation of CD4^+^ T cells (*r*=-0.42) and with the mitochondrial lipid peroxidation level (*r*=-0.34). The number of CD4^+^ T cells and the degree of mitochondrial lipid peroxidation in CD8^+^ T cells likewise showed a negative correlation (*r*=-0.32), and negatively correlated with the secretion of IFN-γ(*r*=-0.31). We also found that the CD4/CD8 ratio was superior to the CD4^+^ T cell count in reflecting T cell mitochondrial functions, especially the mitochondrial lipid peroxidation degree (Fig. 5).

## Discussion

Although ART improves HIV-related clinical symptoms and increases patients’ life expectancies, PLWHs have a significantly increased risk of opportunistic infections and tumors in combination. While decreases in HIV morbidity and mortality are linked to enhancements in CD4^+^ T cell counts resulting from ART, patients treated with ART have a higher risk of pathogenicity and mortalities compared with HIV-uninfected individuals of the same age [2]. This risk can be predicted in part by CD4^+^ T cell counts, but a single metric reveals still less information, requiring clinical discovery of additional potentially valid predictors. So in this study, we conducted a comprehensive assessment of the function of the CD4/CD8 ratio and discovered that it is intimately associated with diverse functions of T lymphocytes in PLWHs.

Firstly, in patients with sustained virological suppression, we discovered a negative connection between activated CD4^+^ (HLA-DR^+^CD38^+^) and CD8^+^ T cells and the CD4/CD8 ratio. A strong relationship between the population of CD4^+^ T cells’ over-activation and exhaustion indicators, namely programmed death-1 (PD-1), and the CD4/CD8 ratio was found by Buggert et al. [28]. The CD4/CD8 ratio is regarded as an indicator of immunological response and immune activation, even in PLWHs undergoing long-term suppressive virus control [20, 29]. This is supported by our research, which showed that in HIV patients with sustained viral suppression, the CD4/CD8 ratio was inversely linked with markers of CD4^+^ T and CD8^+^ T cell activation, suggesting that over-activation of cells affects the restoration of CD4^+^ T cell count as well as the CD4/CD8 ratio.

One of the main features of HIV infection is the metabolic dysfunction of immune cells, an important factor in the pathophysiology and progression of AIDS [30–32]. The ultimate oxidation of sugars, lipids, and amino acids and the release of energy takes place in mitochondria, which is the key mediators of cellular metabolism. Mitochondria are also centres for amino acids, lipids and proteins [33, 34]. The results of the present study showed that mitochondrial lipid peroxidation as well as lipid ROS levels were significantly higher in PLWHs compared with UCs, and were most significant in the LR group, suggesting that HIV infection as well as incomplete recovery of CD4 /CD8 causes mitochondrial oxidative stress and dysfunction. The mitochondrial function of the T cells in the HR group was essentially normal and lipid peroxidative damage was improved. The elevated levels of lipid peroxidation in CD4^+^ T cells in the LR group may be due to the fact that HIV is more prone to infecting cells with high levels of mitochondrial oxidation [35], and also that HIV infection and replication further alters the levels of mitochondrial lipid peroxidation, impairs immune recovery in HIV patients, and increases the risk of sustained replication of residual viruses. Although CD8^+^ T cells cannot be directly infected by viruses, the presence of sustained immune activation and inflammatory responses may still cause high levels of lipid peroxidation. Further analysis of the correlation between mitochondrial lipid peroxidation levels and the CD4/CD8 ratio showed that the lower the CD4/CD8 ratio, the higher the oxidative damage to mitochondria. It was hypothesised that increased levels of mitochondrial lipid peroxidation interact with immune system dysfunction to influence the prognosis of HIV patients. We also found that the CD4/CD8 ratio was significantly better than the CD4^+^ T cell count in mirroring mitochondrial function, which has not been reported in previous studies.

It is believed that injury and infection have a pathological result in inflammation. Recently, inflammation is also becoming more widely accepted as an immune system defense and repair mechanism. Typically, immune activation results in alterations to inflammatory markers and soluble factors [36], including cytokines such as gamma-interferon (IFN-γ), tumor necrosis factor-alpha (TNF-α), interleukin-6 (IL-6), etc [37–40]. Both TNF-α and IFN-γ are secreted by Th1 cells, and TNF-α can mediate the production of inflammatory cytokines, activate organic inflammatory signalling pathways, and induce the synthesis and secretion of IL-6. In this study, we found that with the increase of CD4/CD8 ratio, TNF-α secreted by CD4^+^ T cells in HIV patients gradually decreased, and inflammation was significantly reduced. Our correlation analysis of the CD4/CD8 ratio with indicators of inflammatory activity revealed that the CD4/CD8 ratio was negatively correlated with TNF-α, suggesting that the CD4/CD8 ratio has some potential to present T cell inflammation. This has also been reported in previous studies. According to Keith Sigel et al., low CD4/CD8 ratios are assumed to indicate defective immune activation related to chronic inflammation. They also identified a substantial correlation between the ratio and the pathophysiology of overall T cells [41].

The sample size of this study was small, and the patients were predominantly male and may have gender bias; therefore, the results of this study have some limitations. Further confirmation of the significance of the CD4/CD8 ratio in presenting relevant information within a larger cohort is required. It would be of great interest to figure out whether this ratio has a unique power of presentation compared with that in the general population [42, 43]. In summary, a persistently low CD4/CD8 ratio during the course of effective ART is indicative of ongoing inflammation, mitochondrial malfunction, and immune system failure. Our study highlights the significance of the CD4/CD8 ratio as an indicator of mitochondrial lipid peroxidation for assessing the severity of mitochondrial damage and responses to ART in HIV patients.

## Conclusions

The CD4/CD8 ratio was not superior to the CD4^+^ T cell count in reflecting T cell activation, proliferation, and inflammation. Meanwhile, the amount of mitochondrial lipid peroxidation in T cells was strongly linked with the CD4/CD8 ratio, and was superior to CD4^+^ T cell count in displaying mitochondrial functions. In summary, the CD4/CD8 ratio is a crucial indicator of mitochondrial damage and ART efficacy in PLWHs. It not only has the potential to inform more precise therapeutic interventions but also underlines the necessity of comprehensive immune surveillance, aiming to enhance patient care and ensure favorable long-term health prognosis.

## Data Availability

The raw data supporting the conclusions of this article will be made available by the authors without undue reservation.
